# Extended adjuvant aromatase inhibition after sequential endocrine therapy in postmenopausal women with breast cancer: follow-up analysis of the randomised phase 3 DATA trial

**DOI:** 10.1016/j.eclinm.2023.101901

**Published:** 2023-03-20

**Authors:** Vivianne C.G. Tjan-Heijnen, Senna W.M. Lammers, Sandra M.E. Geurts, Ingeborg J.H. Vriens, Astrid C.P. Swinkels, Carolien H. Smorenburg, Maurice J.C. van der Sangen, Judith R. Kroep, Hiltje de Graaf, Aafke H. Honkoop, Frans L.G. Erdkamp, Wilfred K. de Roos, Sabine C. Linn, Alexander L.T. Imholz, Dutch Breast Cancer Research Group (BOOG) for the DATA Investigators

**Affiliations:** a Department of Medical Oncology, Maastricht University Medical Centre, GROW, Maastricht University, Maastricht, the Netherlands; b Clinical Research Department, Netherlands Comprehensive Cancer Organisation (IKNL), Nijmegen, the Netherlands; c Department of Medical Oncology, Netherlands Cancer Institute, Amsterdam, the Netherlands; d Department of Radiation Oncology, Catharina Hospital, Eindhoven, the Netherlands; e Department of Medical Oncology, Leiden University Medical Centre, Leiden, the Netherlands; f Department of Medical Oncology, Medical Centre Leeuwarden, Leeuwarden, the Netherlands; g Department of Medical Oncology, Isala Clinics, Zwolle, the Netherlands; h Department of Medical Oncology, Zuyderland Medical Centre Heerlen-Sittard-Geleen, Location Sittard-Geleen, Geleen, the Netherlands; i Department of Surgery, Gelderse Vallei Hospital, Ede, the Netherlands; j Department of Pathology, University Medical Centre Utrecht, Utrecht, the Netherlands; k Department of Medical Oncology, Deventer Hospital, Deventer, the Netherlands

**Keywords:** Breast cancer, Adjuvant, Aromatase inhibitor, Endocrine therapy, Extended treatment

## Abstract

**Background:**

The DATA study evaluated the use of two different durations of anastrozole in patients with hormone receptor-positive breast cancer who were disease-free after 2–3 years of tamoxifen. We hereby present the follow-up analysis, which was performed after all patients reached a minimum follow-up of 10 years beyond treatment divergence.

**Methods:**

The open-label, randomised, phase 3 DATA study was performed in 79 hospitals in the Netherlands (ClinicalTrials.gov, number NCT00301457). Postmenopausal women with hormone receptor-positive breast cancer who were disease-free after 2–3 years of adjuvant tamoxifen treatment were assigned to either 3 or 6 years of anastrozole (1 mg orally once a day). Randomisation (1:1) was stratified by hormone receptor status, nodal status, HER2 status, and prior tamoxifen duration. The primary outcome was adapted disease-free survival, defined as disease-free survival from 3 years after randomisation onwards. Adapted overall survival was assessed as a secondary outcome. Analyses were performed according to the intention-to-treat design.

**Findings:**

Between June 28, 2006, and August 10, 2009, 1912 patients were randomly assigned to 3 years (n = 955) or 6 years (n = 957) of anastrozole. Of these, 1660 patients were eligible and disease-free at 3 years after randomisation. The 10-year adapted disease-free survival was 69.2% (95% CI 55.8–72.3) in the 6-year group (n = 827) and 66.0% (95% CI 62.5–69.2) in the 3-year group (n = 833) (hazard ratio (HR) 0.86; 95% CI 0.72–1.01; p = 0.073). The 10-year adapted overall survival was 80.9% (95% CI 77.9–83.5) in the 6-year group and 79.2% (95% CI 76.2–81.9) in the 3-year group (HR 0.93; 95% CI 0.75–1.16; p = 0.53).

**Interpretation:**

Extended aromatase inhibition beyond 5 years of sequential endocrine therapy did not improve the adapted disease-free survival and adapted overall survival of postmenopausal women with hormone receptor-positive breast cancer.

**Funding:**

AstraZeneca.

**Research in context**

**Evidence before this study**

At the start of our study in June 2006, six trials had shown that treatment with an aromatase inhibitor, either upfront for 5 years or sequentially after 2–3 years of tamoxifen for a total endocrine therapy duration of 5 years, was superior to 5 years of tamoxifen monotherapy in postmenopausal women with hormone receptor-positive breast cancer (ATAC, BIG 1–98, IES, ITA, ABCSG-8, and ARNO-95). Furthermore, four other trials had shown that extending the treatment duration of the initial 5 years of tamoxifen with either an additional 5 years of tamoxifen or 5 years of an aromatase inhibitor resulted in an improved outcome (aTTom, ATLAS, MA.17, and NSABP B-33). However, at that point in time, the benefit of extending endocrine therapy with an aromatase inhibitor in postmenopausal women with hormone receptor-positive breast cancer who had already received an aromatase inhibitor as part of the first 5 years of adjuvant endocrine therapy was unknown.

**Added value of this study**

The randomised, phase III DATA study evaluated whether 6 years of aromatase inhibitor therapy is more effective than 3 years of aromatase inhibitor therapy in postmenopausal women with hormone receptor-positive breast cancer who remained free of disease recurrence after 2–3 years of adjuvant tamoxifen, showing no improvement in both disease-free survival and overall survival.

In contrast to the DATA study, both the GIM4 study and the NSABP B-42 study showed a statistically significant disease-free survival benefit of extended aromatase inhibitor therapy in postmenopausal women who had been treated with an aromatase inhibitor as part of their first 5 years of endocrine treatment. The effect sizes for the disease-free survival benefit of all three studies (DATA, GIM4, and NSABP B-42) were however comparable.

**Implications of all the available evidence**

Extended aromatase inhibition may produce a disease-free survival benefit in postmenopausal women who already received 5 years of endocrine therapy that included an aromatase inhibitor. The size of this disease-free survival benefit in the entire population of postmenopausal women with hormone receptor-positive breast cancer, however, remains to be defined. A meta-analysis including all studies on extended aromatase inhibition might provide additional insight into this overall benefit, and might furthermore provide additional insight into the benefit of extended aromatase inhibition in specific subgroups of patients.

## Introduction

Hormone receptor-positive breast cancer is the most common subtype of breast cancer in women, accounting for 75% of all breast cancers.^1^ Although the relative survival has increased over the past decades and is considered rather good, patients remain at risk of recurrence for many years after diagnosis.^2^^,^^3^ In patients who received 5 years of adjuvant endocrine therapy without signs of disease recurrence, the 20-year risk of developing distant recurrences ranged from 13% for patients with T1 disease and no lymph nodes involved to 41% for patients with T2 disease and 4 to 9 nodes involved.^3^ Furthermore, more than half of all distant recurrences occurred beyond the first 5 years of endocrine therapy.

Considering the long-term risk of recurrence, several studies have evaluated the use of extended endocrine therapy beyond 5 years of treatment.^4^, ^5^, ^6^, ^7^, ^8^, ^9^, ^10^, ^11^, ^12^ In patients who received 5 years of adjuvant tamoxifen, extended endocrine therapy with either tamoxifen or an aromatase inhibitor resulted in an improved outcome.^4^, ^5^, ^6^ In postmenopausal women, aromatase inhibitors, either upfront or sequentially after 2–3 years of tamoxifen for a total duration of 5 years, have shown to improve 10-year breast cancer and all-cause mortality rates by an average absolute benefit of 2% when compared with 5 years of tamoxifen.^13^ However, in patients who already received an aromatase inhibitor as part of the initial 5 years of endocrine therapy, the benefit of extended treatment with an aromatase inhibitor still remained to be elucidated. Therefore, trials investigating the extended use of aromatase inhibitors in this group of patients were initiated.^7^, ^8^, ^9^, ^10^, ^11^, ^12^

One of these studies was the DATA study.^7^ In the DATA study, postmenopausal women with hormone receptor-positive breast cancer with no signs of disease recurrence after 2–3 years of adjuvant tamoxifen were randomly assigned to either 3 or 6 years of anastrozole treatment. Of 1860 eligible patients, 1660 were disease-free at 3 years after randomisation, corresponding to the moment of treatment divergence. At the primary analysis, the 5-year adapted disease-free survival beyond treatment divergence was 83.1% in the 6-year treatment group and 79.4% in the 3-year treatment group with a hazard ratio (HR) of 0.79 (95% confidence interval (CI) 0.62–1.02; p = 0.066).

Here, we present the follow-up analysis of the DATA study, which was performed after all patients reached a minimum adapted follow-up of 10 years beyond treatment divergence, corresponding to 13 years beyond date of randomisation and about 15–16 years beyond date of primary breast cancer diagnosis.

## Methods

### Study design and participants

The DATA study was an open-label, randomised, phase 3 trial that enrolled postmenopausal women with hormone receptor-positive early breast cancer from 79 hospitals in the Netherlands between June 2006 and August 2009.^7^ Patients were eligible if they had received 2–3 years of tamoxifen without signs of disease recurrence. Tumours were defined hormone receptor-positive when a positive nuclear staining of at least 10% of the oestrogen or progesterone receptors was present. Patients were allowed to receive (neo) adjuvant chemotherapy and/or radiotherapy. Of note, most patients were diagnosed prior to the implementation of adjuvant trastuzumab in the Netherlands (i.e. September 2005).

The following exclusion criteria were applied: a diagnosis of invasive breast cancer within 10 years before diagnosis of the current breast cancer, previous or concurrent invasive malignancies within 5 years (except for squamous or basal cell carcinoma of the skin or carcinoma in situ of the cervix), a Karnofsky performance score of less than 60%, treatment with another study drug, and being unable to adhere to the trial regimen.

The study was performed under the auspices of the Dutch Breast Cancer Research Group (BOOG). Approval of the protocol was obtained from the central medical ethics committee, located in the Radboud University Medical Centre (Nijmegen, the Netherlands), and local hospital boards. The study was performed in agreement with the principles of the Declaration of Helsinki and Good Clinical Practice guidelines. All patients gave written informed consent. The full study protocol is available online (https://clinicaltrials.gov/ProvidedDocs/57/NCT00301457/Prot_000.pdf). Data management was executed by The Netherlands Comprehensive Cancer Organisation (IKNL), an independent central data office. The quality and progression of the study protocol were monitored by an independent data safety monitoring board. The trial was initially sponsored by AstraZeneca. From November 2016 onwards, the Maastricht University Medical Centre (Maastricht, the Netherlands) sponsored the trial. Database lock was on March 7, 2022.

### Randomisation and masking

Patients were randomly allocated (1:1) to either 6 years (extended treatment group) or 3 years (control group) of adjuvant anastrozole. Randomisation was performed centrally using The Trans European Network for Clinical Trials Services (TENALEA). The following stratification factors were included: nodal status, hormone receptor status, Human Epidermal growth factor Receptor-2 (HER2) status, and tamoxifen treatment duration. None of the study participants, treating physicians, or investigators were masked to treatment allocation.

### Procedures

After 2–3 years of tamoxifen, patients received either 3 or 6 years of adjuvant anastrozole at a dose of 1 mg orally once a day. This resulted in a total adjuvant endocrine treatment duration of 5–6 years or 8–9 years, respectively. Dose reductions were not allowed. Follow-up visits were planned twice a year during the first 6 years after randomisation, and yearly thereafter. During those visits, patients were monitored for breast cancer recurrence through history taking and physical examination. In addition, a mammogram was performed once a year.

### Outcomes

The primary outcome was adapted disease-free survival, defined as disease-free survival from 3 years after randomisation onwards since all patients received anastrozole during the first 3 years. A period of disease-free survival ended when one of the following events occurred: (non-)invasive (local, regional, or distant) breast cancer recurrence, second primary (non-)invasive breast cancer, other cancers (excluding squamous or basal cell carcinoma of the skin or carcinoma in situ of the cervix), and death from any cause.

Secondary outcomes included overall survival, breast cancer-free interval, and breast cancer-specific mortality. A period of overall survival ended at the date of death from any cause and a period of breast cancer-specific mortality ended following breast cancer-related death. Breast cancer-free interval events included (local, regional, or distant) breast cancer recurrence and second primary (non-)invasive breast cancer. Just like the primary outcome, all secondary outcomes were evaluated from 3 years after randomisation onwards, providing ‘adapted’ outcomes. In the absence of an event, patients were censored at the last follow-up visit. Safety and compliance results have been reported previously.^7^

### Statistical analysis

This study was designed to detect an increase in adapted disease-free survival from 90% in the 3-year treatment group to 94% in the 6-year treatment group at 6 years after randomisation, corresponding to a HR of 0.60.^7^ Considering a statistical power of 80.0% and a two-sided significance level of 0.05, we required 770 disease-free study participants at 3 years after randomisation. As the interim analysis was skipped (Protocol amendment 4), the final analysis was assessed at a significance level of 0.05 and a power of 82.5%. Primary and secondary outcomes were analysed according to the intention-to-treat design, excluding patients who had a disease-free survival event or were lost to follow-up during the first 3 years after randomisation.

The median follow-up time was calculated using the reverse Kaplan–Meier approach. Adapted disease-free survival and overall survival were evaluated using Kaplan–Meier survival analyses. Breast cancer-specific mortality and breast cancer-free interval were assessed with competing risk methodology. Death not related to breast cancer was considered a competing event in the analysis of breast cancer-specific mortality. Death from any cause and second primary non-breast cancer were included as competing events in the analysis of breast cancer-free interval. Differences between the two treatment groups were analysed using the stratified log-rank test and stratified Cox regression analyses. Additionally, we used a stratified Cox regression model to test for interaction of treatment with a risk factor, including the following risk factors: the stratification factors (nodal status, hormone receptor status, HER2 status, and tamoxifen treatment duration), additional prognostic factors (age, tumour size, histology, and grade), and previous use of (neo) adjuvant chemotherapy. An interaction term for treatment and time was also included in the model to test the proportionality assumption.

All statistical analyses were performed using SPSS (version 25) and STATA (version 17). P-values were two-sided and deemed statistically significant at a p-value of less than or equal to 0.05. This trial is registered with ClinicalTrials.gov, number NCT00301457 (other study ID numbers: D5392NL003 and EUDRACT 2005-006167-31).

### Role of the funding source

AstraZeneca was involved in the design and monitoring of the trial. The funding source had no role in data collection, data analysis, data interpretation, and writing of the report. Authors had full access to data and were jointly responsible for interpretation of the data.

## Results

**Fig. 1:**
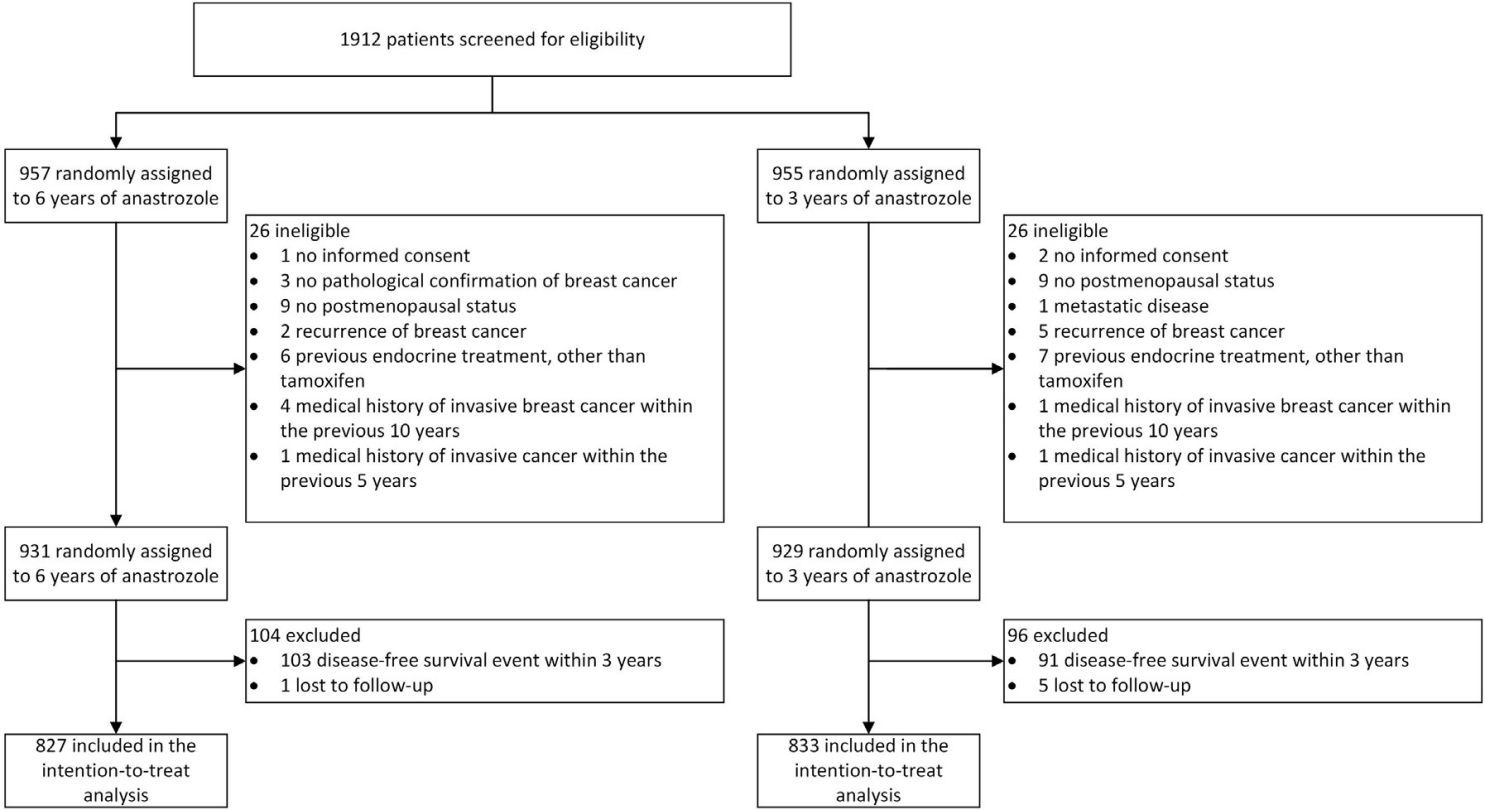
Trial profile.

Between June 28, 2006, and August 10, 2009, 1912 patients were recruited and randomly assigned to 3 years (n = 955) or 6 years (n = 957) of anastrozole (Fig. 1). After exclusion of 52 patients who did not meet the inclusion criteria, the eligible study population consisted of 1860 patients (929 in the 3-year group versus 931 in the 6-year group). Of these, 1660 patients were disease-free at 3 years after randomisation and included in the intention-to-treat analyses. Complete follow-up information was available for 1574 patients (95% of the total study population). Among the 86 patients who were lost to follow-up, 36 had an adapted follow-up period of more than 7 years (i.e. 10 years beyond date of randomisation).

Baseline characteristics were comparable between the treatment groups (Table 1). The majority of patients were younger than 60 years of age at randomisation (58% in the 6-year group versus 59% in the 3-year group), had node-positive disease (68% versus 66%), and were diagnosed with tumours expressing both oestrogen and progesterone receptors (76% in both treatment groups). In 369 (92%) of 400 patients with tumours expressing only one hormone receptor, the progesterone receptor was the absent receptor.

**Table 1:** Baseline characteristics of 1660 patients who were disease-free at 3 years after randomisation.

Characteristic^a^	6-year anastrozole (N = 827)	3-year anastrozole (N = 833)
Median age – years (IQR)	57 (51–64)	57 (51–64)
Age at randomisation – no. (%)		
<60 years	483 (58)	488 (59)
≥60 years	344 (42)	345 (41)
Pathological tumour status – no. (%)		
T1	376 (45)	383 (46)
T2	392 (47)	382 (46)
T3/4	58 (7)	67 (8)
Unknown	1 (<1)	1 (<1)
Pathological nodal status – no. (%)		
Negative	266 (32)	282 (34)
Positive	561 (68)	551 (66)
Tumour grade – no. (%)		
G1	139 (17)	158 (19)
G2	430 (52)	415 (50)
G3	229 (28)	238 (29)
Unknown	29 (4)	22 (3)
Hormone receptor status – no. (%)		
ER+/PR+	627 (76)	633 (76)
ER+/PR-	188 (23)	181 (22)
ER-/PR+	12 (2)	19 (2)
HER2 status – no. (%)		
Positive	18 (2)	22 (3)
Negative	745 (90)	748 (90)
Unknown	64 (8)	63 (8)
Histology – no. (%)		
Ductal	606 (73)	636 (76)
Breast-conserving surgery – no. (%)		
Yes	433 (52)	408 (49)
Prior (neo) adjuvant chemotherapy – no. (%)		
Yes	565 (68)	570 (68)
Previous use of tamoxifen – no. (%)		
≤2.5 years	606 (73)	598 (72)
>2.5 years	221 (27)	235 (28)

Percentages may exceed 100% because of rounding.

ER = oestrogen receptor, HER2 = human epidermal growth factor receptor 2, IQR = interquartile range, PR = progesterone receptor.

a There were no statistically significant differences in baseline characteristics between the groups.

**Fig. 2:**
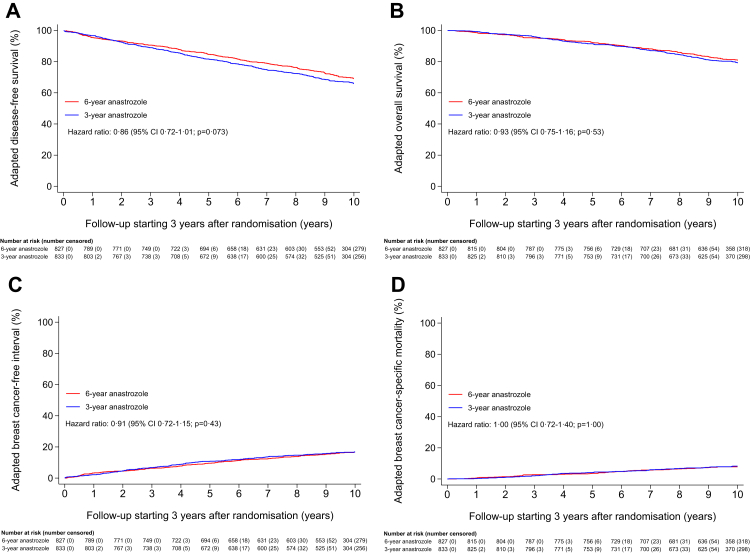
Adapted disease-free survival (A), adapted overall survival (B), adapted breast cancer-free interval (C), and adapted breast cancer-specific mortality (D) in 1660 patients who were disease-free at 3 years after randomisation.

**Fig. 3:**
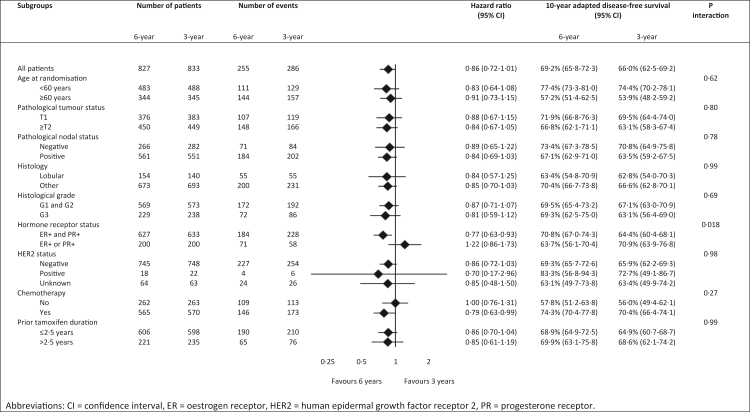
Explorative subgroup analyses of adapted disease-free survival comparing 6 years of anastrozole with 3 years of anastrozole.

After a median adapted follow-up period of 10.1 years (IQR 9.5–10.8) beyond treatment divergence, 541 patients had developed a disease-free survival event and 335 patients had died. Details about primary and secondary outcome events are presented in Table 2. No statistically significant difference in adapted disease-free survival was observed between treatment groups: the 10-year adapted disease-free survival was 69.2% (95% CI 65.8–72.3) in the 6-year treatment group and 66.0% (95% CI 62.5–69.2) in the 3-year treatment group (HR 0.86; 95% CI 0.72–1.01; p = 0.073) (Figs. 2A and 3, Supplementary Table S2).

**Table 2:** Efficacy outcome events of the 1660 patients who were disease-free at 3 years after randomisation.

Event	Number of patients (%)	
	6-year anastrozole (N = 827)	3-year anastrozole (N = 833)
**Primary outcome**		
Adapted disease-free survival event^a^	255	286
Recurrence of the primary tumour	111 (44)	113 (40)
Local recurrence	19 (7)	15 (5)
Regional recurrence	21 (8)	22 (8)
Distant recurrence^b^	92 (36)	90 (31)
Visceral	49 (19)	54 (19)
Bone	56 (22)	57 (20)
Soft tissue	18 (7)	13 (5)
Other	4 (2)	3 (1)
Second, (non-)invasive breast cancer	27 (11)	34 (12)
Ipsilateral	6 (2)	6 (2)
Ipsilateral invasive	5 (2)	4 (1)
Ipsilateral DCIS	0 (0)	2 (1)
Other	1 (<1)	0 (0)
Contralateral	21 (8)	29 (10)
Invasive	14 (5)	23 (8)
DCIS	5 (2)	6 (2)
Other	2 (1)	0 (0)
Second, non-breast cancer^c^	64 (25)	86 (30)
Death without prior breast cancer event	56 (22)	58 (20)
**Secondary outcome**		
Death from any cause	163	172
Breast cancer related	70 (43)	69 (40)
Not breast cancer related	93 (57)	103 (60)
Second primary malignancy	29 (18)	37 (22)
Cardiovascular disease	15 (9)	19 (11)
Other	49 (30)	47 (27)

DCIS = ductal carcinoma in situ.

a Patients may have had multiple disease-free survival events at the same moment.

b Multiple locations of distant recurrence were reported in some patients.

c Additional information about type of second primary, non-breast cancer is presented in Supplementary Table S1.

The 10-year adapted overall survival was not statistically significantly different between the treatment groups: 80.9% (95% CI 77.9–83.5) in the 6-year treatment group and 79.2% (95% CI 76.2–81.9) in the 3-year treatment group (HR 0.93; 95% CI 0.75–1.16; p = 0.53) (Fig. 2B, Supplementary Fig. S1, Supplementary Table S2).

No statistically significant difference in adapted breast cancer-free interval was observed between treatment groups. The 10-year adapted cumulative incidence of breast cancer-free interval events was 16.6% (95% CI 14.1–19.3) in the 6-year treatment group and 16.8% (95% CI 14.3–19.5) in the 3-year treatment group (HR 0.91; 95% CI 0.72–1.15; p = 0.43) (Fig. 2C).

The 10-year adapted cumulative incidence of breast cancer-specific mortality was also not statistically significantly different between treatment groups: 7.8% (95% CI 6.1–9.8) in the 6-year treatment group and 8.2% (95% CI 6.5–10.3) in the 3-year treatment group (HR 1.00; 95% CI 0.72–1.40; p = 1.00) (Fig. 2D).

**Fig. 4:**
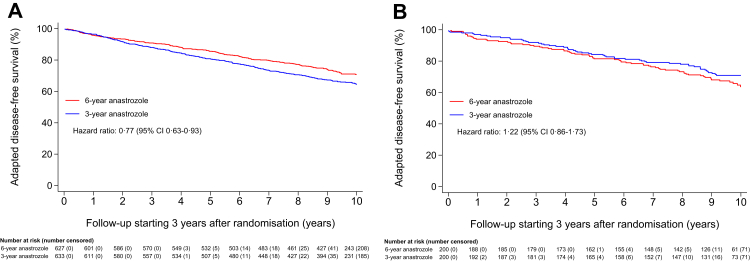
Adapted disease-free survival in (A) patients diagnosed with an oestrogen receptor- and progesterone receptor-positive tumour, and (B) patients diagnosed with an oestrogen receptor- or progesterone receptor-positive tumour.

The explorative subgroup analyses of adapted disease-free survival are presented in Fig. 3. In patients with tumours expressing both the oestrogen and progesterone receptor, a statistically significant benefit of 6.4% favouring 6 years of anastrozole was observed, with a 10-year adapted disease-free survival of 70.8% (95% CI 67.0–74.3) in the 6-year treatment group versus 64.4% (95% CI 60.4–68.1) in the 3-year treatment group (HR 0.77; 95% CI 0.63–0.93) (Figs. 3 and 4A, Supplementary Table S2). In patients with tumours expressing only one hormone receptor, this benefit was not observed: the 10-year adapted disease-free survival was 63.7% (95% CI 56.1–70.4) in the 6-year treatment group and 70.9% (95% CI 63.9–76.8) in the 3-year treatment group (HR 1.22; 95% CI 0.86–1.73) (Figs. 3 and 4B). This difference in treatment effects by hormone receptor status, which was a stratification factor, was shown to be statistically significant (p interaction = 0.018) (Fig. 3). Treatment effects were similar in the other subgroups (Fig. 3).

The effect of extended aromatase inhibition on the adapted overall survival was not statistically significantly different between patients with tumours expressing both the oestrogen and progesterone receptor and patients with tumours expressing only one hormone receptor (p interaction = 0.051) (Supplementary Fig. S1). In patients with tumours expressing both the oestrogen and progesterone receptor, the 10-year adapted overall survival was 82.7% (95% CI 79.4–85.5) in the 6-year treatment group and 78.7% (95% CI 75.2–81.8) in the 3-year treatment group (HR 0.83; 95% CI 0.65–1.07) (Supplementary Figs. S1 and S2A, Supplementary Table S2). Patients with tumours expressing only one hormone receptor did not experience any benefit in adapted overall survival when receiving extended aromatase inhibition: the 10-year adapted overall survival was 75.2% (95% CI 68.2–80.8) in the 6-year treatment group and 81.0% (95% CI 74.7–86.0) in the 3-year treatment group (HR 1.33; 95% CI 0.86–2.05) (Supplementary Figs. S1 and S2B).

Additional explorative post-hoc subgroup analyses were performed in patients with tumours expressing both the oestrogen and progesterone receptor (Supplementary Fig. S3A and B, Supplementary Fig. S4A and B, Supplementary Table S2). For both adapted disease-free survival and adapted overall survival, the absolute benefit of extended aromatase inhibition increased significantly when additional high-risk factors, i.e. lymph node-positive disease and tumours of larger size, were present. For example, in patients with node-positive tumours of larger size (≥pT2) expressing both the oestrogen and progesterone receptor (26% of included patients), 6 versus 3 years of anastrozole resulted in an absolute improvement of 13.2% in the 10-year adapted disease-free survival (HR 0.64; 95% CI 0.47–0.88) (Supplementary Fig. S3B, Supplementary Table S2).

## Discussion

The DATA study shows that, after a median adapted follow-up of 10.1 years beyond treatment divergence, extended treatment with 3 additional years of anastrozole after 5–6 years of sequential endocrine therapy did not statistically significantly improve the primary outcome adapted disease-free survival (+3.2%, HR 0.86, p = 0.073) in postmenopausal women with hormone receptor-positive breast cancer. Interestingly, however, in our study the hormone receptor status appeared to be a significant predictive factor for the presence of a benefit of extended aromatase inhibition. This was shown by a statistically significant improvement in adapted disease-free survival (+6.4%, HR 0.77) in patients with tumours expressing both oestrogen and progesterone receptors receiving extended treatment, whereas no benefit was observed in patients with tumours expressing only one hormone receptor (p interaction = 0.018). The hormone receptor status, which was a stratification factor in the DATA study, may therefore be considered when prescribing extended aromatase inhibition.

The only other study with a similar design as the DATA study is the GIM4 study from Italy.^12^ At an adapted follow-up of about 9 years beyond treatment divergence, the GIM4 study showed that extended treatment with 2–3 additional years of letrozole after 5 years of sequential endocrine therapy statistically significantly improved the 10-year adapted disease-free survival (+9%, HR = 0.73; 95% CI 0.60–0.90; p = 0.0022) of postmenopausal women with hormone receptor-positive breast cancer. The results of the GIM4 study differ from the results of our study, in which extended aromatase inhibition did not improve the adapted disease-free survival of postmenopausal women with hormone receptor-positive breast cancer who received 5 years of sequential endocrine therapy. It should, however, be noticed that the direction and size of reported HR's of both studies are in line. Small differences in patient characteristics may account for the observed differences between the studies. Specifically, the 10-year adapted disease-free survival was 66.0% in the control group of the DATA study, whereas it was only 59.0% in the control group of the GIM4 study. The 10-year adapted disease-free survival rates were similar in the intervention groups (i.e. 69.2% in the DATA study and 68.0% in the GIM4 study). We believe that a meta-analysis, including all trials on extended aromatase inhibition, might help to define the benefit of extended aromatase inhibition in all postmenopausal women with hormone receptor-positive breast cancer. We furthermore believe that a meta-analysis may be useful to define the benefit of extended aromatase inhibition in specific subgroups of patients. We are looking forward to find out whether our results regarding the predictive impact of the hormone receptor status can be confirmed in a meta-analysis.

To assess the optimal endocrine treatment duration, it is important to address three additional studies on extended aromatase inhibition in patients who already received 5 years of endocrine therapy.^8^, ^9^, ^10^, ^11^ The NSABP B-42 study compared 5 years of extended letrozole with 5 years of placebo treatment in postmenopausal women who were disease-free after 5 years of adjuvant endocrine therapy with either an aromatase inhibitor or tamoxifen followed by an aromatase inhibitor.^9^^,^^10^ The IDEAL and ABCSG16 studies compared 2-2.5 years with 5 years of extended aromatase inhibition therapy in postmenopausal women who were disease-free after 5 years of adjuvant endocrine therapy with either tamoxifen, an aromatase inhibitor, or tamoxifen followed by an aromatase inhibitor.^8^^,^^11^ The NSABP B-42 study showed that the 10-year disease-free survival rate statistically significantly improved in patients treated with extended letrozole (+4%, HR 0.84), and this effect was more pronounced in patients who had previously received tamoxifen versus those who had received an aromatase inhibitor upfront (HR 0.74 versus HR 0.90).^10^ Extended letrozole therapy did not improve the overall survival. The IDEAL study and ABCSG16 study both indicated that 5 years of extended aromatase inhibition was not superior to 2-2.5 years of extended aromatase inhibition.^8^^,^^11^ Altogether, these studies suggest that in (selected) postmenopausal women with hormone receptor-positive breast cancer who have received 5 years of sequential endocrine therapy the most optimal treatment duration of extended aromatase inhibition is on average 3 years, corresponding to a total endocrine treatment duration of 8 years.

The absolute benefit of extended aromatase inhibition may vary between subgroups of patients. Lymph node status and tumour size are anatomic prognostic factors that can easily be used to identify patients at highest risk of recurrence, i.e. patients who will most likely experience the highest absolute benefit of extended aromatase inhibition.^3^^,^^14^^,^^15^ In addition, predictive factors may be of added value. The ASCO biomarkers guideline for early-stage breast cancer recommends to use the Breast Cancer Index (BCI) to guide decisions about extended endocrine therapy.^16^ BCI is a dichotomous index combining two gene expression assays: HOXB13: IL17BR (H: I) and molecular grade index (MGI). BCI was first identified as an independent prognostic factor for late distant recurrence in patients with hormone receptor-positive disease.^17^^,^^18^ Subsequently, studies showed that the predictive component of the BCI, the BCI (H: I), may be useful to identify patients experiencing the highest benefit of extended endocrine therapy.^19^, ^20^, ^21^, ^22^ In these studies, extended endocrine therapy statistically significantly improved outcomes in BCI (H: I)-high patients, whereas extended endocrine therapy did not improve outcomes in BCI (H: I)-low patients. So far, we have not tested the performance of the BCI in our cohort.

Interestingly, the DATA study showed that patients with tumours expressing only one hormone receptor did not benefit from extended endocrine treatment (HR 1.22; 95% CI 0.86–1.73), whereas patients with tumours expressing both hormone receptors did experience a large benefit (HR 0.77; 95% CI 0.63–0.93) (p interaction = 0.018). The absent hormone receptor was in 92% of patients the progesterone receptor. Similar findings were observed in the GIM4 study (HR 1.06 versus HR 0.77 for one receptor versus both receptors present) and the IDEAL study (HR 1.03 and HR 0.63, respectively), although the p interaction tests were not statistically significant in these studies.^8^^,^^12^ The NSAPB B-42 study did not provide details on the influence of the hormone receptor status.^9^^,^^10^ Already 50 years ago, researchers hypothesized that the progesterone receptor, in addition to the oestrogen receptor alone, might be of help in predicting which patients will respond to endocrine therapy.^23^ More recently, the progesterone receptor status was validated as an independent predictive factor for benefit of endocrine therapy with either tamoxifen or an aromatase inhibitor.^24^, ^25^, ^26^ It has long been assumed that the absence of the progesterone receptor reflects a non-functional oestrogen receptor. A recent review by Tokunaga and colleagues, however, suggested that expression of the progesterone receptor is not inhibited via a non-functional oestrogen receptor, but via the PI3K/Akt/mTOR pathway instead.^27^ Activation of this pathway correlates to growth factor signalling. Therefore, a negative progesterone receptor status may function as a measure for endocrine resistance. In this study, we have shown that the progesterone receptor status is a predictive factor for benefit of extended aromatase inhibition therapy.

Apart from extending adjuvant endocrine therapy, other strategies may be beneficial in reducing the risk of recurrence in (postmenopausal) patients with hormone receptor-positive breast cancer. These include chemotherapy and cyclin-dependent kinase (CDK) 4/6 inhibitors in patients with a high–risk profile, HER2-targeted therapy in patients with HER2-positive disease, poly ADP ribose polymerase (PARP) inhibitor therapy in patients with germline BRCA1/2 mutations, and use of bone-modifying agents. In addition, several trials currently test the value of next generation oral selective oestrogen receptor degraders (SERDs). These new approaches will likely improve the outcome of future patients diagnosed with hormone receptor-positive breast cancer, though it should be recognised that the additional value of each treatment may decline because of the lower residual risk of relapse.

A major strength of our study is the inclusion of a heterogeneous group of postmenopausal women with hormone receptor-positive breast cancer, as a result of broad eligibility criteria. Therefore, our results are applicable to a wide range of postmenopausal women with hormone receptor-positive breast cancer. Our study also has some limitations. We did not register toxicities in this current follow-up analysis. Long-term toxicities were however reported in the prior final analysis of the DATA study and other studies on extended aromatase inhibition, concluding that the small increased risk of cardiovascular disease and bone fractures should be considered in the decision-making process.^7^^,^^28^ We also did not collect tissue from locoregional or distant recurrences. Much may still have to be learned from studying endocrine resistance after different durations of aromatase inhibition. For instance, the impact of the development of ESR1 mutations following extended aromatase inhibition needs to be clarified. Furthermore, one may propose that the use of results from subgroup analyses is another limitation of our study. However, we believe that, even though the main results of our study did not reach statistical significance, the observed results in subgroups may still be relevant for clinical practice. A meta-analysis including all studies on extended aromatase inhibition, which is soon expected to be done by the Early Breast Cancer Trialists’ Collaborative Group (EBCTCG), will hopefully provide additional insight in which subgroups of patients benefit the most from extended aromatase inhibition.

In this study, extended aromatase inhibition did not improve the adapted disease-free survival and overall survival of postmenopausal women with hormone receptor-positive breast cancer who received 5 years of sequential endocrine therapy. We therefore do not advice to extend treatment with an aromatase inhibitor beyond 5 years of sequential endocrine therapy in all postmenopausal women with hormone receptor-positive breast cancer. An interesting finding of our study, however, is that the effect of extended aromatase inhibition on adapted disease-free survival differed between patients with tumours expressing both the oestrogen and progesterone receptor and patients with tumours expressing only one hormone receptor. This finding warrants further study.

## Contributors

The study was designed and performed under the auspices of the Dutch Breast Cancer Research Group (BOOG). VCGT-H, SWML, SMEG, IJHV, CHS, MJCvdS, and JRK contributed to conceptualisation. Data management was executed by the Netherlands Comprehensive Cancer Organisation (IKNL), Nijmegen, the Netherlands (under the guidance of ACPS). VCGT-H, ACPS, CHS, JRK, HdG, AHH, FLGE, WKdR, SCL, and ALTI contributed to resources. VCGT-H, SWML, SMEG, IJHV, and ACPS accessed and verified the raw data. SMEG was responsible for all statistical analyses. VCGT-H, SWML, SMEG, and IJHV interpreted the study results and prepared the initial manuscript. All authors provided feedback on the first draft of the manuscript. After careful considerations of provided feedback, the final manuscript was prepared by VCGT-H, SWML, SMEG, and IJHV. All authors gave approval for publication of the final manuscript.

## Data sharing statement

The study protocol is available at https://clinicaltrials.gov/ProvidedDocs/57/NCT00301457/Prot_000.pdf. The individual participant data will be shared with the Early Breast Cancer Trialists’ Cooperative Group. Other researchers who are interested in using data are invited to send a methodologically sound proposal to vcg.tjan.heijnen@mumc.nl. After approval of the proposal, data will be made available to the researchers. Data will be available for the next 5 years following the date of Article publication.

## Declaration of interests

VCGT-H reports grants and personal fees from AstraZeneca during the conduct of the study and outside the submitted work; grants and personal fees from Novartis and Lilly; grants from Roche, Pfizer, Daiichi Sankyo, and Gilead outside the submitted work. VCGT-H has a consulting role for AstraZeneca, Lilly, and Novartis. SWML reports grants from AstraZeneca during the conduct of the study; grants from Lilly outside the submitted work. SMEG reports grants from AstraZeneca during the conduct of the study; grants from Roche, Pfizer, Novartis, Lilly, Daiichi Sankyo, and Gilead; personal fees from AstraZeneca outside the submitted work. IJHV reports grants from AstraZeneca during the conduct of the study; grants from Pfizer and Lilly outside the submitted work. ACPS and ALTI report grants from AstraZeneca during the conduct of the study. CHS is chair of the Board of Dutch national breast cancer guidelines. JRK reports grants from AstraZeneca, MSD, Eisai, Lilly, and GSK outside the submitted work. JRK has been a Data Safety Monitoring Board or Advisory Board member for the Alison trial (UMCG) and the TEIPP trial (EMC). AHH reports grants from the Dutch Breast Cancer Research Group during the conduct of the study and outside the submitted work. AHH has been an Advisory Board member for Lilly. AHH received support from Pfizer to attend the ESMO 2022 congress. SCL reports grants from AstraZeneca during the conduct of the study and outside the submitted work; grants from Eurocept Plaza, Roche, Genentech, Gilead Sciences, Tesaro, Novartis, Dutch Cancer Society, ZonMW, A Sister's Hope [Z]aan de Wandel, and Agendia outside the submitted work; consulting fees from AstraZeneca, ERC (EU), and NWO (Dutch Research Council); payment or honoraria from Daiichi Sankyo; other financial support for attending meetings from Daiichi Sankyo, ESMO, ERC (EU), and NWO (Dutch Research Council); non-financial support from Genentech (drug), Roche (drug), Gilead Sciences (drug), Novartis (drug), Agendia (gene expression tests), and AstraZeneca (drug). SCL has a patent (UN23A01/P-EP) pending on a method for assessing homologous recombination deficiency in ovarian cancer cells. SCL is chair of the Trial Steering Committee of the PIONEER trial (Cambridge University) and Member of the Health Council of the Netherlands – Independent scientific advisory body for government and parliament. The other authors have declared no conflicts of interests.

## Appendix A Supplementary data

Supplementary_appendix_DATA study_20230216

Study protocol DATA study
