# Supplementary material for: Extended adjuvant aromatase inhibition after sequential endocrine therapy in postmenopausal women with breast cancer: follow-up analysis of the randomised phase 3 DATA trial
Source: eClinicalMedicine. 2023 Mar 20;58:101901. doi: 10.1016/j.eclinm.2023.101901 (PMC10041456; doi:10.1016/j.eclinm.2023.101901)
Supplement: Supplementary_appendix_DATA study_20230216 [file mmc1.pdf]

Supplementary appendix

Supplement to: Tjan-Heijnen V.C.G., Lammers S.W.M., Geurts S.M.E., et al. Extended adjuvant aromatase inhibition after sequential endocrine therapy in postmenopausal women with breast cancer: follow-up analysis of the randomised phase 3 DATA trial.

Acknowledgments

This study was funded by AstraZeneca. We would like to thank all treating physicians, trial site principal investigators, and local data managers who contributed to the recruitment, treatment, and follow-up of study participants. In addition, we would like to thank Irene van Hellemond, who was extensively involved in the primary analysis of the DATA study. Furthermore, we acknowledge the contributions from Petronella Peer and Wim Lemmens (Radboud University Medical Centre, Netherlands) who conducted all statistical analyses for the primary analysis of the DATA study.

Table of contents

Supplementary Table 1. Type of second, non-breast cancers in 1660 patients who were disease-free at 3 years after randomisation 3

Supplementary Figure 1. Explorative subgroup analyses of adapted overall survival comparing 6 years of anastrozole with 3 years of anastrozole 4

Supplementary Figure 2. Adapted overall survival in (A) patients diagnosed with an oestrogen receptor- and progesterone receptor-positive tumour, and (B) patients diagnosed with an oestrogen receptor- or progesterone receptor-positive tumour 5

Supplementary Figure 3. Adapted disease-free survival in (A) patients diagnosed with an oestrogen receptor- and progesterone receptor-positive, node-positive tumour, and (B) patients diagnosed with an oestrogen receptor- and progesterone receptor-positive, node-positive tumour of larger size (≥pT2) 6

Supplementary Figure 4. Adapted overall survival in (A) patients diagnosed with an oestrogen receptor- and progesterone receptor-positive, node-positive tumour, and (B) patients diagnosed with an oestrogen receptor- and progesterone receptor-positive, node-positive tumour of larger size (≥pT2) 7

Supplementary Table 2. Adapted disease-free survival and adapted overall survival in subgroups of patients 8

CONSORT 2010 Checklist DATA study 9

Supplementary Table 1. Type of second, non-breast cancers in 1660 patients who were disease-free at 3 years after randomisation

	Number of patients (%)	
	6-year anastrozole (N=827)	3-year anastrozole (N=833)
Second, non-breast cancer	64	86
Gastrointestinal cancer	21 (33)	21 (24)
Colorectal cancer	10 (16)	13 (15)
Liver cancer	0 (0)	1 (1)
Oesophageal cancer	4 (6)	1 (1)
Pancreatic cancer	2 (3)	5 (6)
Stomach cancer	4 (6)	1 (1)
Other	1 (2)	0 (0)
Genitourinary cancer	4 (6)	9 (10)
Bladder cancer	3 (5)	6 (7)
Renal cancer	1 (2)	3 (3)
Gynaecologic cancer	7 (11)	10 (12)
Cervical cancer	0 (0)	1 (1)
Endometrial cancer	4 (6)	6 (7)
Ovarian cancer	3 (5)	2 (2)
Other	0 (0)	1 (1)
Head & Neck cancer	2 (3)	3 (3)
Hematologic malignancies	8 (13)	8 (9)
Lung cancer	11 (17)	18 (21)
Melanoma	5 (8)	9 (10)
Sarcoma	3 (5)	3 (3)
Other	3 (5)	5 (6)

Supplementary Figure 1. Explorative subgroup analyses of adapted overall survival comparing 6 years of anastrozole with 3 years of anastrozole

Subgroups	Number of patients		Number of events		Hazard ratio (95% CI)	10-year adapted overall survival (95% CI)		P interaction
	6-year	3-year	6-year	3-year		6-year	3-year	
All patients	827	833	163	172	0·93 (0·75-1·16)	80·9% (77·9-83·5)	79·2% (76·2-81·9)	
Age at randomisation								0·46
<60 years	483	488	60	56	1·06 (0·73-1·53)	88·4% (85·1-91·0)	88·8% (85·6-91·3)	
≥60 years	344	345	103	116	0·90 (0·69-1·18)	70·2% (64·8-74·9)	65·6% (60·0-70·6)	
Pathological tumour status								0·60
T1	376	383	66	66	1·00 (0·71-1·42)	83·4% (79·1-86·9)	82·7% (78·3-86·3)	
≥T2	450	449	97	105	0·88 (0·67-1·17)	78·7% (74·5-82·3)	76·4% (72·1-80·2)	
Pathological nodal status								0·51
Negative	266	282	44	55	0·83 (0·56-1·24)	83·7% (78·3-87·9)	80·9% (75·7-85·1)	
Positive	561	551	119	117	0·98 (0·76-1·26)	79·5% (75·8-82·7)	78·4% (74·6-81·7)	
Histology								0·91
Lobular	154	140	39	35	0·93 (0·57-1·52)	75·6% (67·5-82·0)	74·2% (65·8-80·8)	
Other	673	693	124	137	0·92 (0·72-1·18)	82·1% (78·9-84·8)	80·3% (77·0-83·1)	
Histological grade								0·74
G1 and G2	569	573	104	109	0·94 (0·71-1·23)	81·9% (78·4-84·9)	80·8% (77·2-84·0)	
G3	229	238	50	58	0·85 (0·58-1·25)	79·6% (73·6-84·4)	75·7% (69·6-80·7)	
Hormone receptor status								0·051
ER+ and PR+	627	633	114	135	0·83 (0·65-1·07)	82·7% (79·4-85·5)	78·7% (75·2-81·8)	
ER+ or PR+	200	200	49	37	1·33 (0·86-2·05)	75·2% (68·2-80·8)	81·0% (74·7-86·0)	
HER2 status								0·64
Negative	745	748	141	153	0·91 (0·72-1·14)	81·2% (78·1-83·9)	79·6% (76·4-82·4)	
Positive	18	22	3	4	0·82 (0·14-4·74)	88·9% (62·4-97·1)	81·6% (58·0-92·7)	
Unknown	64	63	19	15	1·22 (0·62-2·43)	74·4% (61·5-83·4)	74·5% (61·3-83·8)	
Chemotherapy								0·82
No	262	263	80	83	0·98 (0·71-1·34)	69·0% (62·7-74·5)	67·3% (60·9-72·9)	
Yes	565	570	83	89	0·91 (0·67-1·23)	86·3% (83·1-88·9)	84·6% (81·3-87·4)	
Prior tamoxifen duration								0·58
≤2·5 years	606	598	120	130	0·90 (0·71-1·16)	80·4% (76·8-83·4)	78·3% (74·7-81·5)	
>2·5 years	221	235	43	42	1·03 (0·67-1·58)	82·3% (76·5-86·8)	81·6% (75·8-86·1)	

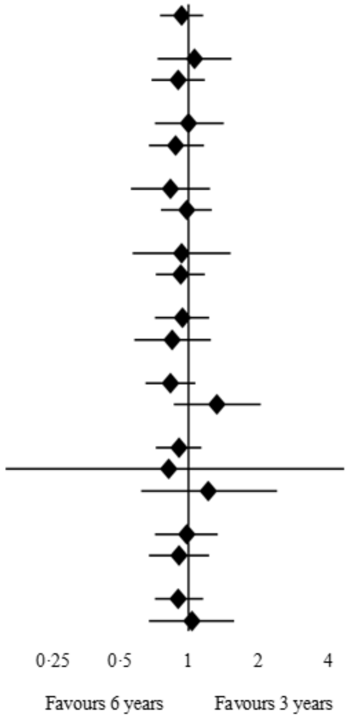

Abbreviations: CI = confidence interval, ER = oestrogen receptor, HER2 = Human Epidermal growth factor Receptor 2, PR = progesterone receptor

Supplementary Figure 2. Adapted overall survival in (A) patients diagnosed with an oestrogen receptor- and progesterone receptor-positive tumour, and (B) patients diagnosed with an oestrogen receptor- or progesterone receptor-positive tumour

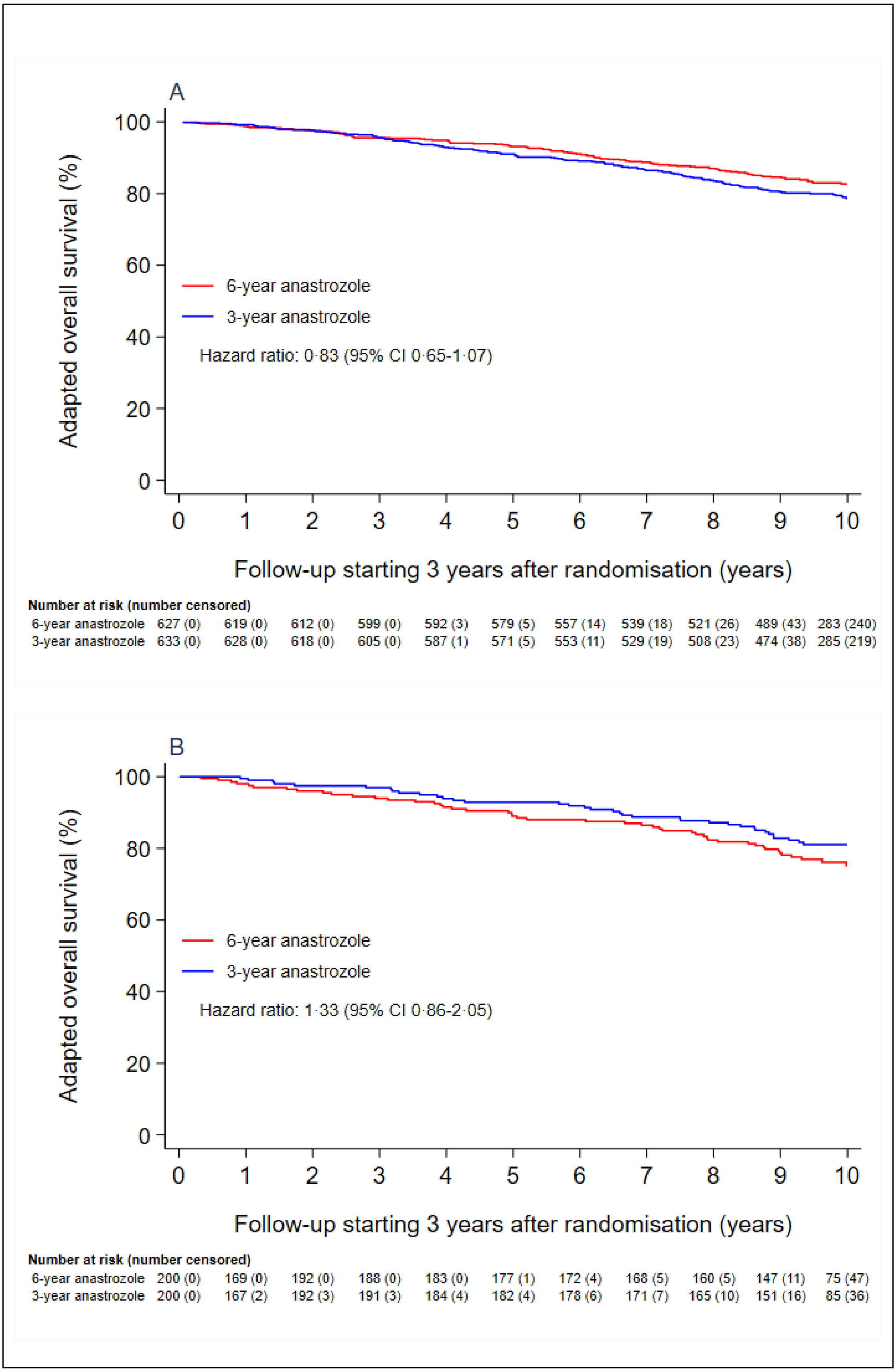

Supplementary Figure 3. Adapted disease-free survival in (A) patients diagnosed with an oestrogen receptor- and progesterone receptor-positive, node-positive tumour, and (B) patients diagnosed with an oestrogen receptor- and progesterone receptor-positive, node-positive tumour of larger size (≥pT2)

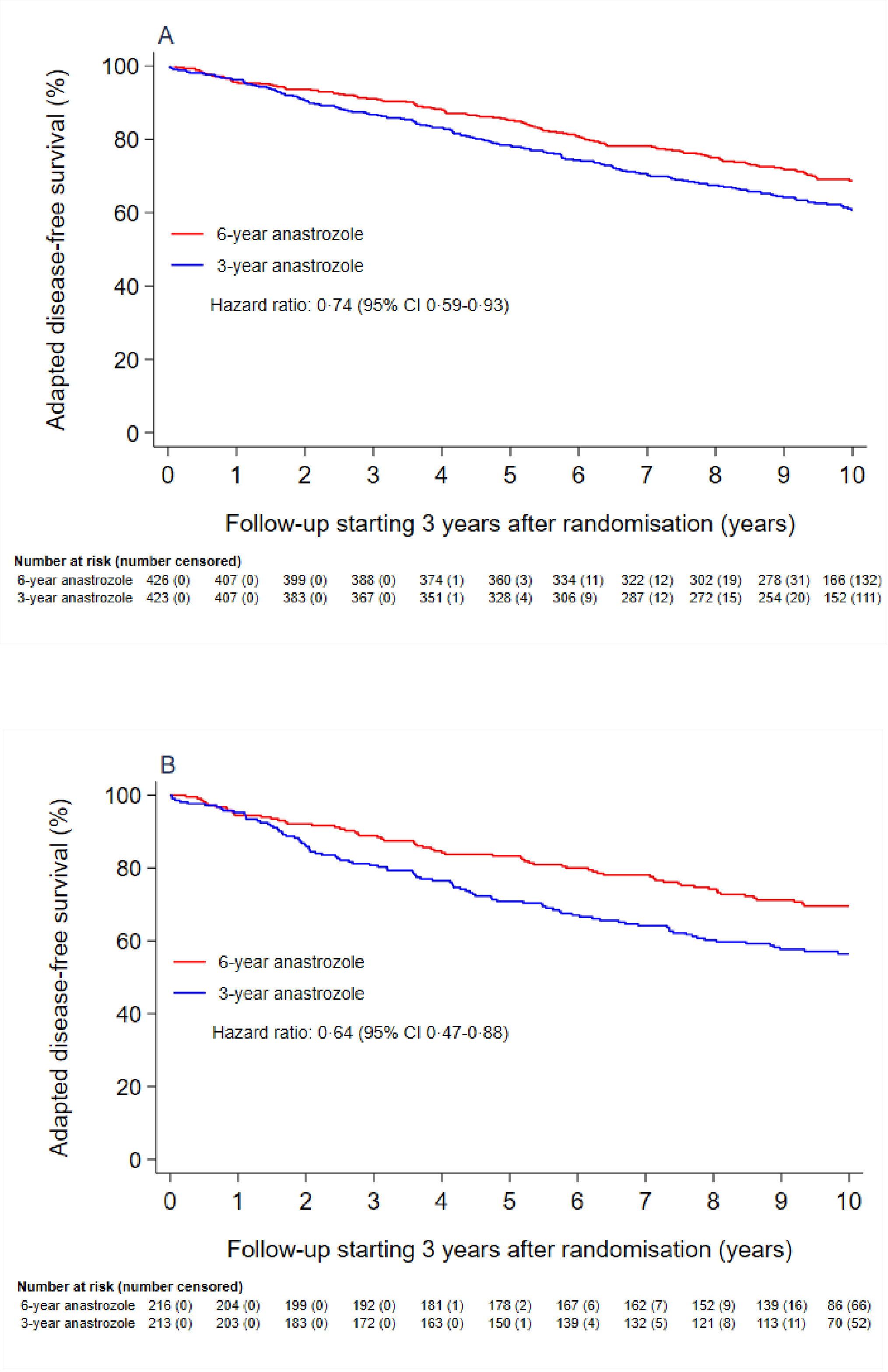

Supplementary Figure 4. Adapted overall survival in (A) patients diagnosed with an oestrogen receptor- and progesterone receptor-positive, node-positive tumour, and (B) patients diagnosed with an oestrogen receptor- and progesterone receptor-positive, node-positive tumour of larger size (≥pT2)

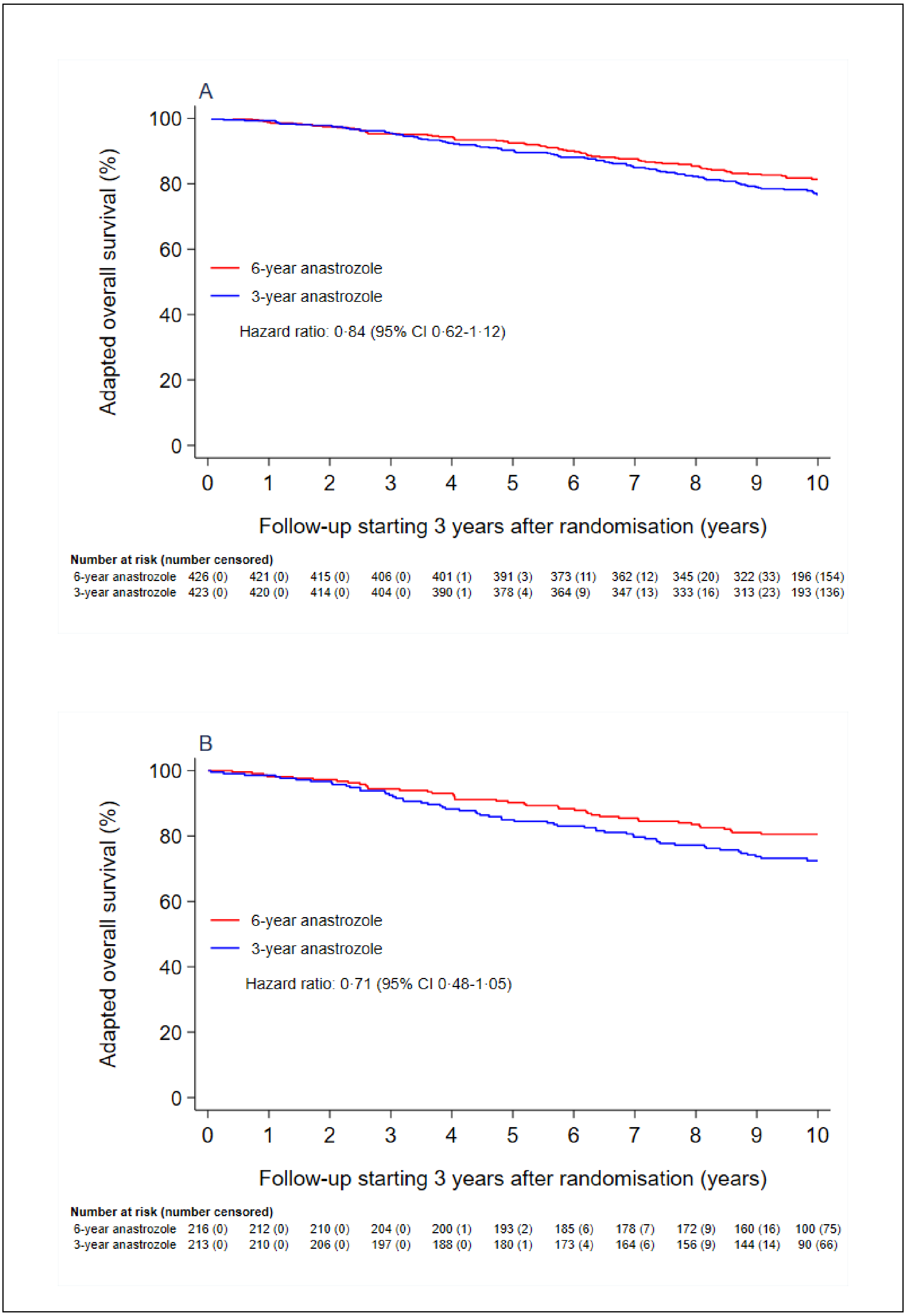

Supplementary Table 2. Adapted disease-free survival and adapted overall survival in subgroups of patients

**Adapted disease-free survival**					
	N = 1660	10-year aDFS (95% CI)		Δ	HR (95% CI)
	% of patients	6-year	3-year		
**All patients**	100%	69·2% (65·8-72·3)	66·0% (62·5-69·2)	3·2%	0·86 (0·72-1·01)
**ER+ and PR+**	76%	70·8% (67·0-74·3)	64·4% (60·4-68·1)	6·4%	0·77 (0·63-0·93)
**ER+ and PR+, pN+**	51%	68·7% (63·9-73·0)	60·7% (55·7-65·3)	8·0%	0·74 (0·59-0·93)
**ER+ and PR+, pN+, ≥pT2**	26%	69·6% (62·8-75·3)	56·4% (49·3-62·8)	13·2%	0·64 (0·47-0·88)
**Adapted overall survival**					
	N = 1660	10-year aOS (95% CI)		Δ	HR (95% CI)
	% of patients	6-year	3-year		
**All patients**	100%	80·9% (77·9-83·5)	79·2% (76·2-81·9)	1·7%	0·93 (0·75-1·16)
**ER+ and PR+**	76%	82·7% (79·4-85·5)	78·7% (75·2-81·8)	4·0%	0·83 (0·65-1·07)
**ER+ and PR+, pN+**	51%	81·4% (77·2-84·9)	76·7% (72·2-80·6)	4·7%	0·84 (0·62-1·12)
**ER+ and PR+, pN+, ≥pT2**	26%	80·6% (74·6-85·3)	72·5% (65·8-78·1)	8·1%	0·71 (0·48-1·05)
Abbreviations: aDFS = adapted disease-free survival, aOS = adapted overall survival, ER+ = oestrogen receptor-positive, HR = hazard ratio, pN+ = node-positive, PR+ = progesterone receptor-positive.					

CONSORT 2010 checklist of information to include when reporting a randomised trial*

Section/Topic	Item No	Checklist item	Reported on page No
**Title and abstract**			
	1a	Identification as a randomised trial in the title	1
	1b	Structured summary of trial design, methods, results, and conclusions (for specific guidance see CONSORT for abstracts)	3-4
**Introduction**			
Background and objectives	2a	Scientific background and explanation of rationale	7-8
	2b	Specific objectives or hypotheses	8
**Methods**			
Trial design	3a	Description of trial design (such as parallel, factorial) including allocation ratio	8-9
	3b	Important changes to methods after trial commencement (such as eligibility criteria), with reasons	11
Participants	4a	Eligibility criteria for participants	8
	4b	Settings and locations where the data were collected	8
Interventions	5	The interventions for each group with sufficient details to allow replication, including how and when they were actually administered	9-10
Outcomes	6a	Completely defined pre-specified primary and secondary outcome measures, including how and when they were assessed	10
	6b	Any changes to trial outcomes after the trial commenced, with reasons	Not applicable

Sample size	7a	How sample size was determined	11
	7b	When applicable, explanation of any interim analyses and stopping guidelines	Not applicable
Randomisation:			
Sequence generation	8a	Method used to generate the random allocation sequence	9
	8b	Type of randomisation; details of any restriction (such as blocking and block size)	9
Allocation concealment mechanism	9	Mechanism used to implement the random allocation sequence (such as sequentially numbered containers), describing any steps taken to conceal the sequence until interventions were assigned	Previously reported (Tjan-Heijnen et al. Lancet Oncol 2017)
Implementation	10	Who generated the random allocation sequence, who enrolled participants, and who assigned participants to interventions	Previously reported (Tjan-Heijnen et al. Lancet Oncol 2017)
Blinding	11a	If done, who was blinded after assignment to interventions (for example, participants, care providers, those assessing outcomes) and how	Not applicable
	11b	If relevant, description of the similarity of interventions	Not applicable
Statistical methods	12a	Statistical methods used to compare groups for primary and secondary outcomes	11-12
	12b	Methods for additional analyses, such as subgroup analyses and adjusted analyses	11-12
**Results**			
Participant flow (a diagram is strongly recommended)	13a	For each group, the numbers of participants who were randomly assigned, received intended treatment, and were analysed for the primary outcome	12
	13b	For each group, losses and exclusions after randomisation, together with reasons	12

Recruitment	14a	Dates defining the periods of recruitment and follow-up	9, 12
	14b	Why the trial ended or was stopped	Not applicable
Baseline data	15	A table showing baseline demographic and clinical characteristics for each group	12-13
Numbers analysed	16	For each group, number of participants (denominator) included in each analysis and whether the analysis was by original assigned groups	12
Outcomes and estimation	17a	For each primary and secondary outcome, results for each group, and the estimated effect size and its precision (such as 95% confidence interval)	13
	17b	For binary outcomes, presentation of both absolute and relative effect sizes is recommended	13
Ancillary analyses	18	Results of any other analyses performed, including subgroup analyses and adjusted analyses, distinguishing pre-specified from exploratory	14-15
Harms	19	All important harms or unintended effects in each group (for specific guidance see CONSORT for harms)	Previously reported (Tjan-Heijnen et al. Lancet Oncol 2017)
**Discussion**			
Limitations	20	Trial limitations, addressing sources of potential bias, imprecision, and, if relevant, multiplicity of analyses	19-20
Generalisability	21	Generalisability (external validity, applicability) of the trial findings	19
Interpretation	22	Interpretation consistent with results, balancing benefits and harms, and considering other relevant evidence	15-20
**Other information**			
Registration	23	Registration number and name of trial registry	12
Protocol	24	Where the full trial protocol can be accessed, if available	9
Funding	25	Sources of funding and other support (such as supply of drugs), role of funders	9, 12

*We strongly recommend reading this statement in conjunction with the CONSORT 2010 Explanation and Elaboration for important clarifications on all the items. If relevant, we also recommend reading CONSORT extensions for cluster randomised trials, non-inferiority and equivalence trials, non-pharmacological treatments, herbal interventions, and pragmatic trials. Additional extensions are forthcoming: for those and for up to date references relevant to this checklist, see www.consort-statement.org.
